# Merocel Surgicel Wrap Technique to Manage Diffuse Epistaxis in Patients with Comorbidities

**DOI:** 10.1155/2020/8272914

**Published:** 2020-03-22

**Authors:** Waleed M. Alshehri, Wafaa M. alwehaibi, Muhammad Wasi Ahmed, Abeer Albathi, Bandar Alqahtani

**Affiliations:** ^1^Otolaryngology Department, King Saud Medical City, Riyadh 12746, Saudi Arabia; ^2^Otolaryngology Department, Prince Sultan Medical Military City, Riyadh 12233, Saudi Arabia

## Abstract

Epistaxis, or nasal bleeding, occurs in over half of the general population. It is caused by various etiological factors and affects both sexes and all age groups. The simplest treatment for a nosebleed is pinching of the ala nasi, referred to as the Hippocratic technique. In this study, we adopted different treatment protocols dependent on the severity of bleeding and assessed the etiology and efficacy of these modalities. This was a prospective study. We recruited 25 patients (24 adults and 1 child) who presented with epistaxis in the ENT departments of two tertiary care hospitals. We evaluated the cause of epistaxis and efficacy of the treatments used. All patients had experienced several episodes of epistaxis and were managed using anterior nasal packing with gauze and ointment or with Merocel packs alone. The incidence of epistaxis was more common in males than in females. It was effectively managed by anterior nasal packing with Surgicel-wrapped Merocel. Patients did not experience further episodes of bleeding following the removal of Merocel and retention of Surgicel in place.

## 1. Introduction

Epistaxis is one of the most common ENT emergencies and has been reported in 60% of the general population [1]. It is more common in males than in females [2]. Its incidence shows a bimodal distribution, with peaks at <10 and >50 years of age. Epistaxis, whether spontaneous or otherwise, is experienced by 2 of 3 people in their lifetime; however, only 6% of people require medical treatment and 1.6 in 10,000 require hospitalization [3]. The principal of nasal packing for epistaxis has changed since Hippocrates used sheep's wool on pugilistic noses in ancient Greece [4]. Most patients can be treated within an emergency setting; however, some elderly patients may require more intensive treatments and hospital admission. In rare cases, severe epistaxis can lead to death [5, 6]. The nose has a rich vascular supply, derived from both the external and internal carotid arteries. Historically, epistaxis is classified as anterior or posterior with no definite demarcating line. McGarry [7] recently proposed a standardization of bleeding location, with bleeding anterior and posterior to the plane of the pyriform aperture classified as anterior and posterior epistaxis, respectively. In addition, it can be classified as primary or secondary when there is an underlying coagulopathy, such as in patients who are being treated with anticoagulant/antiplatelet medication.

Different treatment options are used for epistaxis dependent on the severity. In minor cases, compression or plugging of the affected nostril with cotton, direct application of pressure for 5–20 minutes, and cold sponging on the forehead could help control the bleeding. Severe and massive bleeding may be managed by different methods, such as anterior nasal packing [8], posterior nasal packing [9], and chemical and electric cauterization of the bleeding point [10]. There is a wide variety of nasal packing techniques available. The most common are Merocel packing, inflatable balloons, Rapid rhino, and petroleum-infused gauze.

Diffuse oozing, multiple bleeding sites, or recurrent bleeding may indicate systemic causes, such as hypertension and coagulopathies. In such cases, the patients' coagulation profile, blood count, blood grouping, and cross matching should be investigated. In cases of clotting factor deficiency and coagulopathies, the hemodynamic stability of the patient should be ensured with fluid replacement, electrolytes, and blood transfusion.

## 2. Case Presentation

Informed consent was obtained from all patients or their guardians for this study. The ethics of this study were reviewed and approved.

This study was conducted at two tertiary care centers. We recruited 25 patients (24 adults and 1 child) who presented with epistaxis with comorbidities. Fifteen patients admitted through the medical units were being treated with anticoagulant therapy, 6 patients were terminally ill or announced DNR (do not resuscitate) when admitted to the Intensive Care Unit, and 4 patients did not agree to admission (Table 1).

Of the 25 patients who presented with epistaxis, 17 were males and 8 females, respectively. The age of the patients ranged from 8 to 100 years. The major causes of epistaxis in these patients were hypertension, cardiac disorder, and coagulopathy. All patients had a history of conventional nasal packing by ribbon gauze or Merocel alone (Table 2).

All patients had experienced several episodes of nasal bleeding from both nostrils and were managed by ribbon gauze or Merocel packing. Each time the packs were removed, the bleeding would restart, both anteriorly and posteriorly. Following nasal suction to remove residual clots, two disposable cotton packs soaked in a local 1 : 1 mixture of 0.1% oxymetazoline and 1% topical lidocaine were inserted into each nostril by direct visualization and maintained in place for a few minutes to control bleeding and identify bleeding sites.

**Figure 1:**
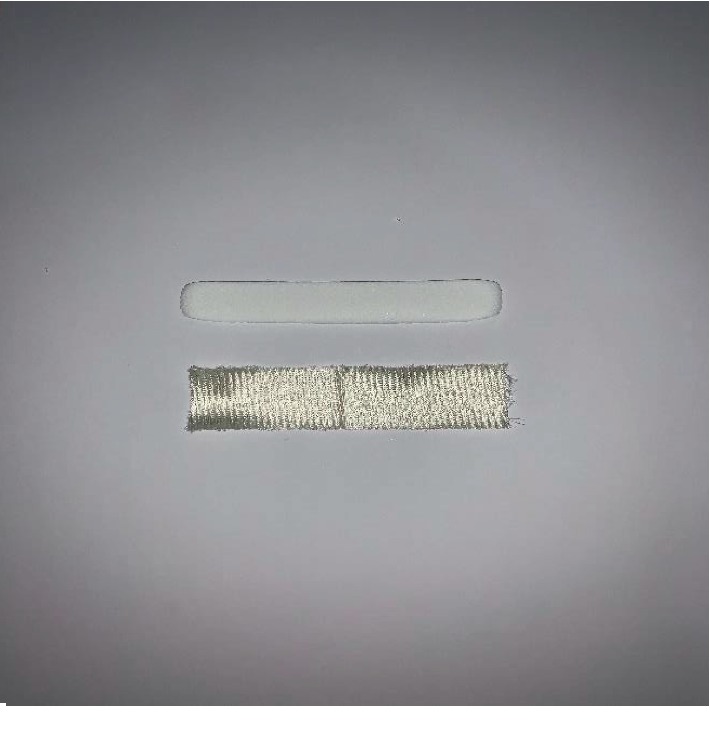
Merocel (above) and Surgicel (below) aligned.

**Figure 2:**
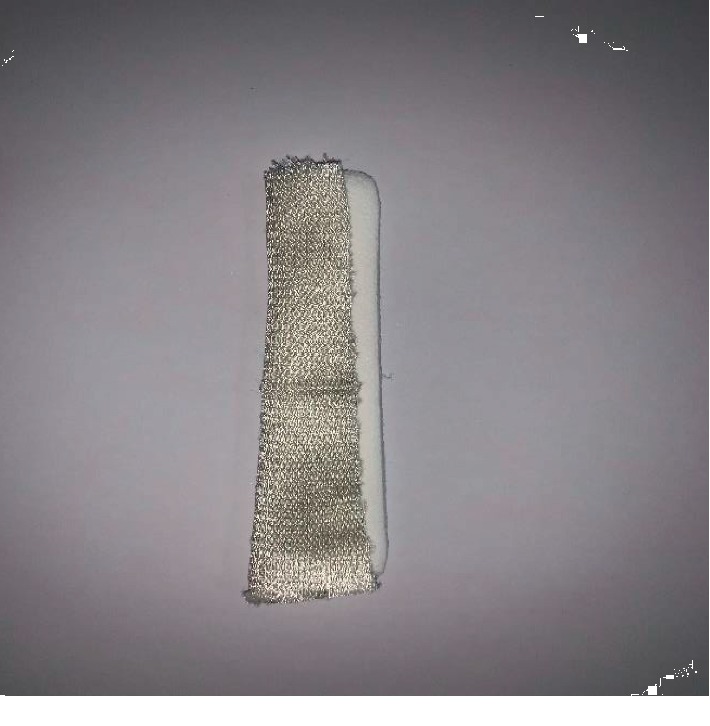
Merocel wrapped with Surgicel and ready for insertion.

Once the cotton pledges were removed, the bleeding began again. Next, we wrapped Merocel (length, 8–10 cm) with Surgicel and inserted it into each nostril of all adult patients. To make sure that the Merocel Surgicel maintains good alignment, make sure to hold the two together with forceps or by lubricating them with gel (Figures 1 and 2). For the child, we wrapped Merocel (length, 4 cm) with Surgicel. Packs were lubricated before insertion, expanded using 10 ml of saline, and inserted according to the manufacturers' instruction. Nasal bleeding and postnasal bleeding were controlled by nasal packing. During nasal packing, we assessed the pain and discomfort associated with the pack *in situ*. The packs were left *in situ* for 48 hours. Four patients who had arrived from remote areas were provided with follow-up 48 hours after nasal pack removal. All patients received prophylactic antibiotics during the treatment duration. In all cases, we removed the Merocel from the nasal packs after 48 hours, leaving behind the Surgicel.

After removal of Merocel packs, no further bleeding was observed. All patients, except the 4 individuals who were from remote areas, were followed up for one week. In all cases, no further episodes of bleeding were observed.

## 3. Discussion

Epistaxis is the most common otorhinolaryngologic emergency that requires hospital admission. Arterial epistaxis is the result of degenerative changes affecting the tunica media [11]. Shaheen suggested local ischemic changes as a potential cause of epistaxis [12]. Epistaxis could be attributed to common local factors, such as digital trauma, septal deviation, chemical irritants, and inflammation or systemic factors, such as coagulopathies, renal failure, alcoholism, and vascular abnormalities [13, 14]. Hypertension is a major cause for epistaxis. It is responsible for 31.82–47.3% of all cases [15–18], which could be linked to anxiety in some patients [19].

Anterior epistaxis is more common in adults, while posterior epistaxis predominates in individuals who are >60 years of age [20]. In addition, anterior epistaxis is the most common condition in ENT practice. This term was first described by Cullen in 1785. To date, multiple theories relating to etiology, clinical manifestations, diagnosis, and management have been reported in the literature [21]. There are a wide variety of nasal packing techniques available, such as ribbon gauze, Bismuth iodine paraffin paste, and balloon catheters. These techniques, although effective, can cause complications, such as patient discomfort, infection, septal perforation, pressure necrosis of nasal alae, and cardiovascular instability. Stangerup et al. used irrigation with hot water as a method of treatment for posterior epistaxis, which was effective and less painful and reduced hospital stay when compared with traditional nasal packs [22].

The ideal nasal pack should provide effective control of epistaxis; smooth insertion and removal; painless use; comfort in place; and minimum risk of aspiration, tissue sensitivity, and infection. However, there is always a risk of aspiration irrespective of the type of nasal pack used [23]. The chance of aspiration or swallowing is small with Merocel because of its size and shape. In addition, it is recommended to secure the string of Merocel to the nasal dorsum or cheek. However, Hashmi et al. [24] reported a case of a nasal pack being swallowed during the treatment of epistaxis, which caused bowel obstruction and perforation.

In our study, bleeding was successfully controlled in 96% of the patients by using Merocel with Surgicel. This rate of success was higher than that in studies by Pringle et al. [25] and Corbridge et al. [8], who reported success rates of 91.5% and 92.6%, respectively, in patients with epistaxis treatment with a Merocel pack alone.

Merocel is a compressed, dehydrated sponge composed of hydroxylated polyvinyl acetate. After insertion, it requires rehydration with normal saline to achieve its optimal size within the nasal cavity and compress the bleeding vessels. In addition, it acts as a surface for platelet aggregation and actively encourages hemostasis. Surgicel is an oxidized regenerated cellulose that has been used clinically for over 5 decades [26–28]. It absorbs water and swells to provide tamponade at the bleeding sites. Its fibers entrap fluid, blood proteins, platelets, and cells to form a gel-like “pseudo-clot” that acts as a barrier to blood flow and subsequently as a matrix for solid fibrin clot formation. Shinkwin et al. evaluated the clinical effectiveness of Surgicel, Vaseline gauze, and Merocel as forms of nasal packing. They concluded that there is less discomfort while using Surgicel both *in situ* and on removal when compared with Vaseline packs and Merocel [29]. Pressure dressing and sutures are commonly used to achieve hemostasis; however, numerous products had been developed to achieve the same aim. These include topical hemostatic agents, such as sponges, thrombin, gelatin-thrombin, fibrin glue, and other types of surgical sealants [30]. Oxidized regenerated cellulose is fully absorbed in 7–14 days with minimal tissue reaction, and it shows antimicrobial activity against a wide range of Gram-positive and Gram-negative organisms, including methicillin-resistant *Staphylococcus aureus* (MRSA) both *in vitro* and *vivo* [31].

In this study, no complications were observed with the use of Merocel or Surgicel. Bleeding was successfully controlled in all patients except one, who exhibited rebleeding. Following removal of the Merocel pack, we kept the Surgicel *in situ* to ensure the formation of a hemostatic plug that would be absorbed eventually. Taken together, our results and previous studies show that absorbable packs are associated with fewer complications. Furthermore, patients with anterior nasal packing can be managed safely as outpatients with no adverse events [32].

With regard to the study limitations, we were unable to blind the physician investigator or patients. Moreover, the follow-up periods were limited; therefore, the long-term outcomes remain unclear.

## 4. Conclusion

Epistaxis is the most common otorhinolaryngological emergency that requires hospitalization. Our results provide evidence that it can be effectively managed with Merocel and Surgicel. To the best of our knowledge, this is the first time the wrap technique has been described.

Anterior nasal packing via Surgicel-wrapped Merocel is safe and effective for recurrent nasal bleeding in patients with comorbidities.

## Figures and Tables

**Table 1:** Patient characteristics.

Patient	Number of patients
Total number of patients	25
Male patients	17
Female patients	8
Adults	24
Children	1
Ward inpatients	15
Intensive care unit patients	6
Outpatients	4

**Table 2:** Patient clinical data.

Pt. number	Age	Sex	Risk factor	Anticoagulation use	Site	Pain (out of 10)	Previous epistaxis	Complication
1	8	Male	None	Yes	Unilateral	N/A	Yes	**Rebleeding**
2	65	Male	HTN	No	Bilateral	6	Yes	**None**
3	72	Male	Cardiac/HTN	Yes	Bilateral	6	Yes	**None**
4	51	Male	Cardiac/HTN	Yes	Bilateral	N/A	Yes	**None**
5	68	Male	DM/HTN	Yes	Bilateral	5	Yes	**Non**
6	48	Male	HTN	Yes	Bilateral	N/A	Yes	**None**
7	76	Male	Cardiac/HTN	Yes	Bilateral	6	Yes	**Non**
8	56	Male	HTN	Yes	Unilateral	7	Yes	**None**
9	100	Male	HTN	Yes	Bilateral	N/A	Yes	**None**
10	19	Female	Renal failure	Yes	Bilateral	7	Yes	**None**
11	53	Female	DM/HTN	Yes	Bilateral	8	Yes	**None**
12	57	Female	Cardiac/HTN	Yes	Bilateral	5	Yes	**None**
13	33	Male	Cardiac	Yes	Bilateral	4	Yes	**None**
14	89	Male	DM/HTN	Yes	Yilateral	6	Yes	None
15	64	Male	Cardiac/HTN	Yes	Bilateral	N/A	Yes	**None**
16	40	Male	Renal failure	Yes	Bilateral	5	Yes	**None**
17	59	Female	DM/HTN	Yes	Unilateral	4	Yes	**None**
18	72	Male	DM/HTN	No	Unilateral	5	Yes	**None**
19	60	Female	DM/HTN	Yes	Bilateral	6	Yes	**None**
20	55	Male	HTN	No	Bilateral		Yes	**None**
21	85	Female	Cardiac DM/HTN	Yes	Bilateral	N/A	Yes	**None**
22	61	Male	HTN	Yes	Bilateral	5	Yes	**None**
23	79	Male	Renal failure	Yes	Unilateral	4	Yes	**None**
24	77	Female	DM/HTN	Yes	Bilateral	6	Yes	**None**
25	58	Female	DM/HTN	Yes	Bilateral	6	Yes	**None**

N/A: for patients either in coma or intubated and sedated; DM: diabetes mellitus; HTN: hypertension.

## Data Availability

The data used to support the findings of this study are available from the corresponding author upon request.

## Additional Points

We were unable to blind the physician investigator or patients in this study. The follow-up times were limited; therefore, the long-term outcomes are unclear.
